# Audiovisual Speech Perception at Various Presentation Levels in Mandarin-Speaking Adults with Cochlear Implants

**DOI:** 10.1371/journal.pone.0107252

**Published:** 2014-09-15

**Authors:** Shu-Yu Liu, Grace Yu, Li-Ang Lee, Tien-Chen Liu, Yung-Ting Tsou, Te-Jen Lai, Che-Ming Wu

**Affiliations:** 1 Institute of Medicine, Chung Shan Medical University, Taichung, Taiwan; 2 School of Speech Language Pathology and Audiology, Chung Shan Medical University, Taichung, Taiwan; 3 Department of Otolaryngology, Chung Shan Medical University Hospital, Taichung, Taiwan; 4 Department of Otolaryngology - Head and Neck Surgery, National University Health System of Singapore, Singapore, Singapore; 5 Department of Otolaryngology, Chang-Gung Memorial Hospital, College of Medicine, Chang-Gung University, Linkou, Taiwan; 6 Department of Otolaryngology, National Taiwan University Hospital, Taipei, Taiwan; 7 Department of Psychiatry, Chung Shan Medical University Hospital, Taichung, Taiwan; Human Brain Research Center, Japan

## Abstract

**Objectives:**

(1) To evaluate the recognition of words, phonemes and lexical tones in audiovisual (AV) and auditory-only (AO) modes in Mandarin-speaking adults with cochlear implants (CIs); (2) to understand the effect of presentation levels on AV speech perception; (3) to learn the effect of hearing experience on AV speech perception.

**Methods:**

Thirteen deaf adults (age = 29.1±13.5 years; 8 male, 5 female) who had used CIs for >6 months and 10 normal-hearing (NH) adults participated in this study. Seven of them were prelingually deaf, and 6 postlingually deaf. The Mandarin Monosyllablic Word Recognition Test was used to assess recognition of words, phonemes and lexical tones in AV and AO conditions at 3 presentation levels: speech detection threshold (SDT), speech recognition threshold (SRT) and 10 dB SL (re:SRT).

**Results:**

The prelingual group had better phoneme recognition in the AV mode than in the AO mode at SDT and SRT (both p = 0.016), and so did the NH group at SDT (p = 0.004). Mode difference was not noted in the postlingual group. None of the groups had significantly different tone recognition in the 2 modes. The prelingual and postlingual groups had significantly better phoneme and tone recognition than the NH one at SDT in the AO mode (p = 0.016 and p = 0.002 for phonemes; p = 0.001 and p<0.001 for tones) but were outperformed by the NH group at 10 dB SL (re:SRT) in both modes (both p<0.001 for phonemes; p<0.001 and p = 0.002 for tones). The recognition scores had a significant correlation with group with age and sex controlled (p<0.001).

**Conclusions:**

Visual input may help prelingually deaf implantees to recognize phonemes but may not augment Mandarin tone recognition. The effect of presentation level seems minimal on CI users' AV perception. This indicates special considerations in developing audiological assessment protocols and rehabilitation strategies for implantees who speak tonal languages.

## Introduction

Verbal information transmitted to listeners via dual-modal (i.e., audiovisual, AV) stimulation is often thought to be more efficient than uni-modal (auditory-only, AO) stimulation [1]–[2]. Listeners, whether hearing-impaired or not, automatically watch talkers' facial, lip and jaw movements, especially when auditory information was degraded, distorted or noise-masked [3]–[5]. In fact, optical cues also provide useful information when auditory stimuli are clear [6]. For example, English listeners distinguish “threat” from “fret” better by observing the location of teeth and tongue of the talkers.

Cochlear implantation has been proven as an effective treatment to restore the hearing of patients with severe-to-profound sensorineural hearing loss [7]. It was reported that deaf patients with cochlear implants (CIs) made use of visual information to supplement the auditory stimulation they received from the CIs and in this way optimized their speech perception in daily communication (e.g., [8]–[10]). Their speech recognition was significantly better in the AV condition than in the AO condition [10]. Higher AV gain was observed in the CI users than in the NH controls who were tested in the simulated or noise-masked conditions as a result of CI users' greater capability to integrate visual information with degraded auditory signals [11].

This AV integration ability in CI users was reported to correlate with the duration of the implant experience rather than the duration of deafness [12]. The neuroplasticity involving speech-related network in our brain seems to allow a more efficient AV integration of speech after cochlear implantation [13]. Yet, although visual speech perceptual skills that developed during periods of deafness could have positive implications for later perception of auditory speech signals [14], visual take-over found in the auditory cortex in some CI users may also lead to incomplete reversal of this deafness-induced cortical reorganization [15]. Due to the inconsistent results from the past studies, the effect of auditory experience on AV perception in CI patients is still in question.

However, although extensive research has been undertaken in non-tonal language users with CIs regarding AV speech processing, little information is available for the patients who speak tonal languages such as Mandarin Chinese. In Mandarin Chinese, each monosyllabic word comprises two lexical components: phoneme(s) and lexical tone. Words could be semantically different solely because of the lexical tone variations. Smith and Burnham [16] and Chen and Massaro [17] were the only ones we found who investigated the tone perception ability of Mandarin-speaking adults in the AV condition. They used normal-hearing participants and focused only on lexical tone discrimination. The authors found that visual information seemed less informative for Mandarin Chinese listeners than for non-tonal language users when discriminating Mandarin tones, meaning that native listeners of Mandarin Chinese depended more on auditory signals than visual ones to distinguish lexical tones.

The presentation levels of speech signals could also affect speech recognition performance in listeners with normal hearing [18] and with CIs [19]–[20]. In general, speech stimuli were more difficult to recognize at soft levels, and listeners often reported to take advantage of visual cues when auditory input was unreliable [3]–[5]. Thus, the degree of dependency on visual cues to distinguish speech stimuli increased with decreasing sensation levels in the normal-hearing listeners [21]. However, the loudness perception of speech stimuli in CI patients can be quite different from normal-hearing ones as they receive sounds through electrical hearing, making their dynamic range much narrower than normal-hearing listeners [20]. Firszt et al.'s study [19] indicated that the CI patients' recognition performances on monosyllabic words and sentences were strongly dependent on presentation levels as their scores decreased consistently when the stimulus level was reduced from 60 to 50 dB SPL. However, it is still unknown whether and how much the CI adults depend on visual information to better recognize the speech stimuli at various presentation levels. In this study, we intend to explore the effect of speech presentation level on visual benefits.

Therefore, the present study aimed (1) to evaluate the recognition performance at word-level, phoneme-level and tone-level in AV mode and in AO mode in Mandarin-speaking Chinese adults with CIs, (2) to understand the effect of presentation levels on their AV speech perception, and (3) to learn the possible effect of hearing experience on AV speech perception in CI listeners.

## Materials and Methods

### Participants

Thirteen (8 male and 5 female) deaf adults with CIs participated in this study (hereafter as the “CI group”, Table 1). They had bilateral severe-to profound sensorineural hearing loss and received unilateral implantation. All of them were recruited from the CI center of Chang-Gung Memorial Hospital, Linkou, Taiwan. No neurological and psychological disorders were found in these subjects, and their verbal intelligence quotient was all higher than 70 (Wechsler Adult Intelligence Scale, 3^rd^ edition) [22]–[23]. They aged between 18.1 years and 56.5 years (mean  =  29.1±13.5; median  = 30.2; interquartile range, IQR  = 23.7) at the time this study took place and had been using the implants for more than 0.5 year (median  = 4.7; IQR  = 6.3). Seven of them were prelingually deafened (before the age of 5 years, the “prelingual group”) and 6 were postlingually deafened (after the age of 5 years, the “postlingual group;” see Table 1). Ten (4 male and 6 female) healthy NH adults were recruited as controls (the “NH group”), aged between 19 years and 26 years (mean  = 21.6±2.9; median  =  20.5; IQR  = 4.8). They did not have any middle ear anomalies or history of otological/neurological diseases. Their hearing thresholds at 500, 1000, 2000 and 4000 Hz were all below 25 dB HL. All of the CI subjects and NH controls were native Mandarin Chinese speakers and had normal or corrected-to-normal vision. The study protocol and written informed consent form was approved by the Institutional Review Board of the Chang Gung Memorial Hospital. All written informed consent forms signed by the participants involved in the present study were obtained before the test procedures took place.

**Table 1:** Demographical background of the prelingually deaf group, the postlingually deaf group and the normal-hearing (NH) group.

		Prelingual		Postlingual		NH	
		N	Median (IQR)	N	Median (IQR)	N	Median (IQR)
Gender (M/F)		3/4		5/1		4/6	
ImpSide (L/R)		4/3		5/1			
Age (y)			17.2 (0.8)		41.2 (6.4)		20.5 (4.8)
OnsetDeaf (y)			4.0 (4.1)		37.9 (9.1)		
DuraDeaf (y)			16.1 (4.4)		0.5 (0.5)		
AgeImp (y)			9.0 (5.1)		38.7 (8.4)		
DuraImp (y)			8.2 (1.7)		1.7 (1.4)		
SDT (dB HL)			25.0 (7.5)		20.0 (3.8)		−5.0 (10.0)
SRT (dB HL)			35.0 (7.5)		35.0 (11.3)		10.0 (0.0)
ImpPTA (dB HL)			30.0 (8.5)		33.5 (3.8)		
NonImpPTA (dB HL)			106.0 (7.5)		107.5 (3.3)		8.0 (6.5)
CI device	Nucleus CI24RE(CA)	5		3			
	Nucleus CI24R(CS)	2		2			
	Nucleus 24M	0		1			
VisionCon	Normal	3		1		2	
	Nearsighted	1		5		8	
	Presbyopic	2		0		0	
	Cataractous	1		0		0	

IQR: Interquartile range; ImpSide: Side of ear with a implant; OnsetDeaf: Onset of deafness; DuraDeaf: Duration of deafness; AgeImp: Age at implantation; DuraImp: Duration of implant use; SDT: Speech detection threshold; SRT: Speech recognition threshold; ImpPTA: Pure tone average of the implanted ear; NonImpPTA: Pure tone average of the non-implanted or normal-hearing ear; VisionCon: Vision condition.

### Test materials

The Mandarin Monosyllablic Word Recognition Test (MMRT), developed by Tsai et al. [24], was used to assess the word recognition ability. A compact disc, offered by the authors of the test, was used as the test material in this study. The test contains standardized-recorded word stimuli, including 6 lists of phonemically balanced monosyllabic words, each with 25 items (i.e., 150 auditory stimuli in total). It has been reported with satisfactory reliability [25].

To measure AV perception of the participating listeners, a video film was recorded specifically by the authors of this study. A male talker produced the test items of MMRT and recorded using a video recording system. The speaking rate of each word was consistent with the auditory output of MMRT. The production of each word began and ended in a closed-mouth, that is, the neutral position. Then, the video film was edited with matched onsets and offsets of auditory stimuli and displayed simultaneously with the auditory signals.

### Test procedures

The test protocol consisted of two sessions: an AO session (i.e., only auditory stimuli were presented to the participants) and an AV session (i.e., the auditory stimuli were presented together with corresponding visual stimuli shown on the displaying screen of a computer). Both sessions took place in a sound-treated booth where a 19-inch LCD monitor was positioned at the participant's eye level at a distance of 1 meter and one loudspeaker at ear level in front (0°) of the participants. The CI group took the test in the CI Center of Chang-Gung Memorial Hospital and the NH group in Chung Shan Medical University. The CI patients did the test with their implanted ear, while the non-implanted ear was not wearing a hearing aid. The NH control group was tested in one ear only. The ear for testing was randomly selected, and the other ear was covered by a TDH-39 headphone set which introduced a masking noise to prevent possible cross-hearing.

The test started with measurements of warble-tone thresholds (at 500, 1000, 2000 and 4000 Hz), speech detection thresholds (SDT) and speech recognition thresholds (SRT) in sound field. Monosyllabic word recognition performances in AV and AO conditions were tested at their SDT, SRT and 10 dB SL above SRT (SRT+10), resulting in a total of 3 presentation levels in each test session. For example, if a participant's SDT was 30 dB HL and SRT 40 dB HL, his/her word recognition performance would be tested at 30, 40, 50 dB HL. We ensured that the subjects felt comfortable with each presentation level. The subjects wrote down each word they heard/saw after each test item was presented to them. The test procedures of the AV session were the same as those in the AO session except that the video stimuli were not presented in the latter condition. To avoid learning effects, the AO session took place one week after the AV session, and the word lists used to test each subject at each of the presentation levels were randomly selected without duplication in each session. Each word was scored based on the accuracy of the phonemes and the lexical tone. For example, if the test item was “ma3 (*horse*),” and the patient responded “ma4 (*scold*)”, he/she would get 0 point for word recognition, 2 point for phoneme recognition, and 0 point for lexical tone recognition.

### Statistical analysis

The descriptive statistics of these variables were presented as median and interquartile ranges because most of the distributions of the variables were not normal. The Kruskal-Wallis H test was implemented to compare the test results of the three groups, and the Mann-Whitney U test was conducted to compare two groups. The Wilcoxon signed-rank test and the Friedman test were used to make within-group comparisons of two or more than two conditions. Relationships between the recognition scores and scoring type (word, tone, phoneme), mode, intensity level and deafness- or implant-related variables – including onset of deafness, duration of deafness, age at implantation and duration of implant use – were assessed using Spearman correlation coefficient with adjustment (age and sex). Statistical analyses were conducted using SPSS software (version 17.0; SPSS; SPSS, Inc., Chicago, IL, USA). A value of *p*<0.05 was considered significant. The Bonferroni correction was used to adjust the *p* values of multiple comparisons; i.e., the differences between the three groups or intensity levels were significant when *p*<α/3 = 0.017.

## Results

### Comparisons between AV and AO conditions at different presentation levels

**Figure 1:**
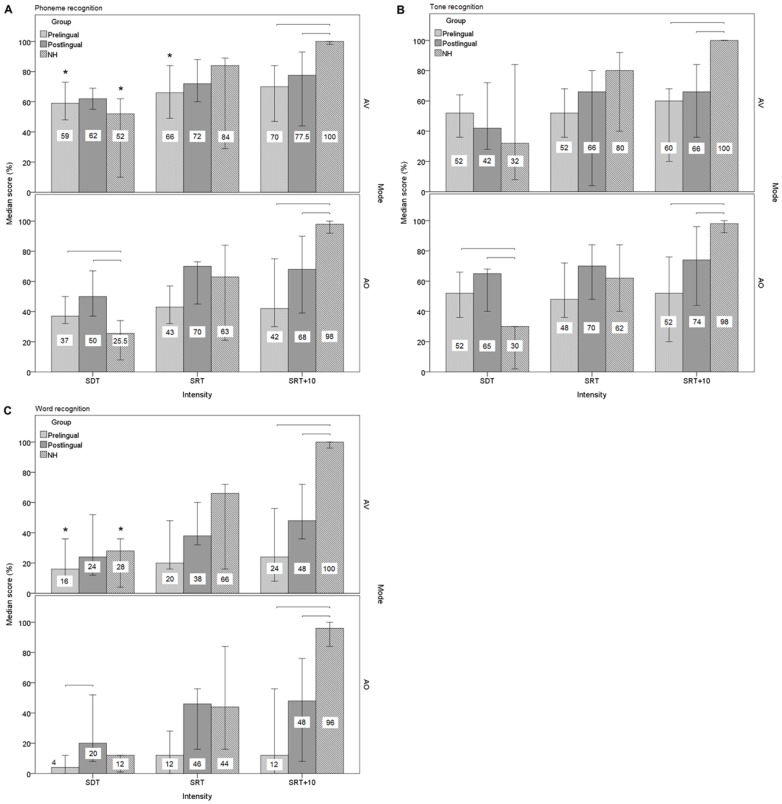
Median recognition scores obtained by the cochlear implanted groups and the normal-hearing group. Median recognition scores of (A) phonemes, (B) lexical tones and (C) words obtained by the prelingual group, the postlingual group and the normal-hearing (NH) group in audiovisual (AV) and auditory-only (AO) modes at the 3 presentation levels. The asterisk marks indicate significant difference between AV and AO modes. The horizontal bars indicate significant difference between groups. The vertical error bars represent 95% confidence interval.

The prelingual group, postlingual group and NH group all had a better phoneme recognition performance in the AV mode than in the AO mode (see Figure 1a). However, the significance was reached only in the prelingual group and NH group (both p<0.001). Using Wilcoxon signed-rank tests with correction for multiple comparisons, the difference between the two modes was significant at SDT and SRT in the prelingual group (both p = 0.016), and at SDT in the NH group (p = 0.004). Significance was not reached at SRT+10 in either group. For tone recognition, none of the three groups had significantly different performances in the two modes (see Figure 1b). Significantly better word recognition performance was noted in the AV mode than in the AO mode in the prelingual group (p<0.001) and the NH group (p = 0.005; see Figure 1c). The significance was reached only at SDT in both groups (p = 0.016 for the prelingual group; p = 0.010 for the NH group).

### Comparisons between groups with different hearing experiences

Using Kruskal-Wallis tests, we found significant differences in phoneme, tone and word recognition between the three groups at SDT and SRT+10 in the AO condition (at SDT, p = 0.001, p<0.001, p = 0.029 respectively for phoneme, tone, word recognition; at SRT+10, all p<0.001). In the AV mode, significance was only reached at SRT+10 (all p<0.001 for phoneme, tone and word recognition). No significant difference was noted at SRT.

Post hoc tests showed that, in the AO mode, the prelingual group and the postlingual group had significantly better phoneme recognition scores than the NH controls at SDT (p = 0.016 and p = 0.002 respectively), while the NH group outperformed the two CI groups at SRT +10 (both p<0.001; see Figure 1a). Similarly for tone recognition, the two CI groups obtained significantly higher scores than the NH group at SDT in the AO mode (p = 0.001 and p<0.001 respectively), and were outperformed by the NH controls at SRT+10 (p<0.001 and p = 0.002 respectively; see Figure 1b). For word recognition, the prelingual group performed worse than the postlingual group at SDT in the AO mode (p = 0.013), and the two CI groups both obtained lower scores than the NH group at SRT+10 (both p<0.001; see Figure 1c). When the test was given in the AV mode, no significant difference was found between the three groups at SDT and SRT. However, at SRT+10, the NH group obtained significantly higher phoneme, tone and word recognition scores than the two CI groups did (all p<0.001; see Figure 1a-c).

### Correlation analysis

The recognition scores had a significant correlation with group when age and sex were controlled (rho  = 0.276, p<0.001). The prelingual group's recognition scores were significantly correlated with scoring type (word, tone or phoneme), mode (AV or AO) and duration of deafness (see Table 2). The postlingual group's recognition scores had significant correlation with scoring type. The recognition scores of the NH group were significantly correlated only with mode and intensity level.

**Table 2:** Spearman correlation coefficients between deafness-related parameters, test conditions and recognition scores.

	Prelingual		Postlingual		NH	
Correlated item	Coefficient*	*p* value	Coefficient*	*p* value	Coefficient*	*p* value
Scoring type^1^	0.657	<0.001	0.542	<0.001	n/s	
Mode^2^	0.237	0.008	n/s		0.152	0.044
Intensity	n/a		n/s		0.840	<0.001
OnsetDeaf	n/a		n/s		n/a	
DuraDeaf	−0.439	<0.001	n/s		n/a	
AgeImp	n/s		n/s		n/a	
DuraImp	n/s		n/s		n/a	

OnsetDeaf: Onset of deafness; DuraDeaf: Duration of deafness; AgeImp: Age at implantation; DuraImp: Duration of implant use; n/s: Not significant; n/a: Not applicable.

*Spearman's correlation coefficients adjusted by sex and age.

1 Scoring type coded as word  = 1, tone  = 2, phoneme  = 3.

2 Mode coded as auditory-only  = 1, audiovisual  = 2.

Only significant correlations are shown.

## Discussion

Researchers have undertaken many studies on audiovisual speech perception in CI users who speak non-tonal languages; however, the performance of the Mandarin-speaking patient group is seldom discussed. Our results indicate that the visual cues from talker's lip and face are informative for phoneme recognition in our prelingually deaf patients with CIs. Yet, vision do not augment Mandarin tone recognition in our CI and NH adults. The presentation level does not affect recognition performance in the CI listeners as much as it does in the NH ones whether in AO or AV mode. The CI users who were deaf at early childhood show poorer speech recognition performance in AV and AO modes and depend more on vision to distinguish phonemes than those patients who were postlingually deafened.

The present study indicates that auditory signals seem to play a major role in identifying lexical tones in Mandarin monosyllabic words for both NH and CI listeners. In other words, visual cues are not found to benefit tone perception at the three intensity levels tested in this study. This finding is similar to Smith and Burnham's study [16] which uses normal-hearing adults and reports that the Mandarin Chinese listeners have worse Mandarin tone recognition scores than the non-tonal Australian English listeners in a visual-only condition. As we know, the pitch variations produced from vocal folds are accessible primarily from audition rather than from vision. However, the signal processing strategies of current CI devices do not transform fundamental frequency of speech stimuli which is important for accurate perception of lexical tones.

On the contrary, visual cues do help phoneme recognition in the prelingual group and the NH group, yet only at threshold levels (i.e., SDT and SRT). It suggests that visual information is required for the NH subjects and the prelingually deaf ones with CIs to recognize phonemes when the auditory information is insufficient. However, the postlingual group performs in a different manner that their phoneme recognition score does not significantly decrease in the absence of visual cues even when the speech intensity is lower than their SRT level. This trend is also found in word recognition that visual cues help the prelingual group and the NH group to better recognize words at SDT, while no significant visual benefit is noted in the postlingual group at any of the intensity levels. The finding that the postlingual group is less dependent on visual information may have some association with their pre-implant language experiences and the automatic gain control provided by the CI. These two factors may allow the postlingual group to show lower dependency on visual information than the prelingual group (who does not have pre-implant hearing experiences) and the NH group (whose acoustic hearing does not adjust loudness input when the intensity level is too low). Because the speech signals experienced by CI patients is transformed by electrical stimulation and the electrical dynamic range of the recipient differs in threshold (T) and comfort (C) levels, microphone sensitivity and volume control, their loudness perception can be quite different from the acoustic hearing perceived by normal listeners. The two CI groups could thus have louder perception at threshold levels than the NH controls. Yet, it also needs to be noted that the median SDT of the NH group is minus 5 dB HL, which does not commonly occur in everyday activities. This may also account for NH controls' lower performance at SDT.

The automatic gain control offered by CI and the narrower dynamic range could make the implantees less sensitive to the changes in the input intensity level as the NH controls are. Unlike the NH subjects whose word recognition performance improves markedly with the increasing sensation levels (scores increased by 72 percentage points from SDT to SRT+10), the CI subjects do not necessarily perform better at higher levels (scores increased by only 8 percentage points in the prelingual group, and by 24 percentage points in the postlingual group; see Figure 1c).

Their lack of sensitivity to the intensity levels in the current study could also be a result that the presentation level at 10 dB SL (re: SRT) is not high enough for them to correctly recognize the word stimuli given the fact that the median presented level at SRT+10 is only 45 dB HL. Therefore, even though the postlingual group manages to score higher than the prelingual group – thanks to their pre-implant hearing experiences – the former still demonstrates worse speech recognition than the NH group. For further studies, degraded sound stimuli (e.g., by using a noise-band vocoder) could be used as the test material for NH controls in order to avoid the ceiling effect observed in our NH subjects and allow a better comparability between CI users and NH listeners. Also, special training on AV integration may be helpful to the CI patients with postlingual deafness as they do not seem to take advantage of the visual information and rely primarily on auditory input even when the acoustic speech signals are barely audible. Yet, further validation using larger sample or data from other institutions is required to test the generalizability of these results.

Furthermore, given that visual information does not help Mandarin tone recognition in our subjects, auditory training programs with a focus on tonal perceptual skills may be helpful for CI users who speak tonal language because lexical tones carry semantic importance and correct lexical tone recognition depends primarily on auditory input. This implies special considerations in developing audiological evaluation protocols and rehabilitation strategies for CI listeners who speak tonal languages.

Some previous studies claim that visual-only lipreading ability deteriorates with age [26]–[27]. Older people may thus gain limited benefit from visual information [28]. However, the result of Cienkowski and Carney [27] study shows that older adults and younger adults actually have similar AV integration ability at syllable level. The poorer visual-only lipreading ability of the older adults does not have a significant influence on successful integration of bisensory information. In the present study, the recognition scores are significantly correlated with group with age and sex controlled, which also implies that age difference is not the cause of the significant between-group difference found in our study. For further studies, visual-only condition is suggested to be taken into consideration to validate the current findings.

Lastly, it should be noted that this study uses monosyllabic words as test materials and that visual cues have different effects on the CI groups' recognition of words, tones and phonemes. It implies that detail analysis of the components of speech signals may help us differentiate the perceptual benefits the implanted devices may provide. Further investigations are required to show the effect of visual information on the CI users when they deal with different forms of speech signals, such as multisyllabic words or sentences.

## Conclusions

Our preliminary results show that vision may help prelingually deaf CI patients to recognize phonemes at threshold presentation levels (i.e., SDT and SRT). However, visual cues may not augment Mandarin tone recognition, at least in our CI and NH subjects. It suggests that auditory training programs with a focus on tonal perceptual skills could be helpful for Mandarin-speaking CI adults to enhance their speech recognition performance as correct perception of tones depends mainly on audition. Moreover, the recognition performance of the CI subjects, whether prelingually or postlingually deafened, does not seem to be significantly affected by the presentation levels regardless of the accessibility of visual cues. These findings indicate special considerations in developing audiological assessment protocols and rehabilitation strategies for CI listeners who speak tonal languages. Further validation is required to test the generalizability of these results, including data from other institutions and other tonal languages.

## References

[1] Grant KW, Walden BE, Seitz PF (1998) Auditory-visual speech recognition by hearing-impaired subjects: consonant recognition, sentence recognition, and auditory-visual integration. J Acoust Soc Am 103: 2677–2690. 9604361 10.1121/1.422788

[2] Arnold P, Hill F (2001) Bisensory augmentation: A speechreading advantage when speech is clearly audible and intact. Br J Psychol 92: 339–355. 11417785

[3] Sumby W, Pollack I (1954) Visual contribution to speech intelligibility in noise. J Acoust Soc Am 26: 212–215.

[4] Sanders DA, Goodrich SJ (1971) The relative contribution of visual and auditory components of speech to speech intelligibility as a function of three conditions of frequency distortion. J Speech Hear Res 14: 154–159. 5550619 10.1044/jshr.1401.154

[5] Massaro DW, Cohen MM (1995) Perceiving talking faces. Current Directions in Psychological Science 4: 104–109.

[6] Campbell R (2008) The processing of audio-visual speech: empirical and neural bases. Philos Trans R Soc Lond B Biol Sci 363: 1001–1010. 17827105 10.1098/rstb.2007.2155 PMC2606792

[7] Waltzman SB (2006) Cochlear implants: current status. Expert Rev Med Devices 3: 647–655. 17064249 10.1586/17434440.3.5.647

[8] Moody-Antonio S, Takayanagi S, Masuda A, Auer ET Jr, Fisher L, et al (2005) Improved speech perception in adult congenitally deafened cochlear implant recipients. Otol Neurotol 26: 649–654. 16015162 10.1097/01.mao.0000178124.13118.76

[9] Kirk KI, Hay-McCutcheon MJ, Holt RF, Gao S, Qi R, et al (2007) Audiovisual Spoken Word Recognition by Children with Cochlear Implants. Audiol Med 5: 250–261. 19823696 10.1080/16513860701673892 PMC2759184

[10] Tyler RS, Fryauf-Bertschy H, Kelsay DM, Gantz BJ, Woodworth GP, et al (1997) Speech perception by prelingually deaf children using cochlear implants. Otolaryngol Head Neck Surg 117: 180–187. 9334763 10.1016/s0194-5998(97)70172-4

[11] Rouger J, Fraysse B, Deguine O, Barone P (2008) McGurk effects in cochlear-implanted deaf subjects. Brain Res 1188: 87–99. 18062941 10.1016/j.brainres.2007.10.049

[12] Desai S, Stickney G, Zeng FG (2008) Auditory-visual speech perception in normal-hearing and cochlear-implant listeners. J Acoust Soc Am 123: 428–440. 18177171 10.1121/1.2816573 PMC2662523

[13] Rouger J, Lagleyre S, Démonet JF, Fraysse B, Deguine O, et al (2012) Evolution of crossmodal reorganization of the voice area in cochlear-implanted deaf patients. Hum Brain Mapp 33: 1929–1940. 21557388 10.1002/hbm.21331 PMC6870380

[14] Mitchell TV, Maslin MT (2007) How vision matters for individuals with hearing loss. Int J Audiol 46: 500–511. 17828666 10.1080/14992020701383050

[15] Sandmann P, Dillier N, Eichele T, Meyer M, Kegel A, et al (2012) Visual activation of auditory cortex reflects maladaptive plasticity in cochlear implant users. Brain 135: 555–568. 22232592 10.1093/brain/awr329

[16] Smith D, Burnham D (2012) Faciliation of Mandarin tone perception by visual speech in clear and degraded audio: implications for cochlear implants. J Acoust Soc Am 131: 1480–1489. 22352518 10.1121/1.3672703

[17] Chen TH, Massaro DW (2008) Seeing pitch: visual information for lexical tones of Mandarin-Chinese. J Acoust Soc Am 123: 2356–2366. 18397038 10.1121/1.2839004 PMC2811545

[18] Beattie RC, Raffin MJ (1985) Reliability of threshold, slope, and PB max for monosyllabic words. J Speech Hear Disord 50: 166–178. 3990262 10.1044/jshd.5002.166

[19] Firszt JB, Holden LK, Skinner MW, Tobey EA, Peterson A, et al (2004) Recognition of speech presented at soft to loud levels by adult cochlear implant recipients of three cochlear implant systems. Ear Hear 25: 375–387. 15292777 10.1097/01.aud.0000134552.22205.ee

[20] Donaldson GS, Allen SL (2003) Effects of presentation level on phoneme and sentence recognition in quiet by cochlear implant listeners. Ear Hear 24: 392–405. 14534410 10.1097/01.AUD.0000090340.09847.39

[21] Binnie CA (1973) Bi-sensory articulation functions for normal hearing and sensorineural hearing loss patients. Journal of Academic Rehabilitation in Audiology 6: 43–53.

[22] Wechsler D (1997) Wechsler Adult Intelligence Scale-Third Edition. San Antonio, TX: The Psychological Corporation.

[23] Chen Y, Chen H (2002) Wechsler Adult Intelligence Scale-Third Edition (Mandarin Version). Taipei: Chinese Behavioral Science Corporations.

[24] Tsai KS, Tseng LH, Wu CJ, Young ST (2009) Development of a mandarin monosyllable recognition test. Ear Hear 30: 90–99. 19125031 10.1097/AUD.0b013e31818f28a6

[25] Wu CJ (2009) Reliability of the Mandarin Monosyllable Recognition Test in Hearing-Impaired Listeners. MA Thesis, National Taipei University of Nursing and Health Sciences, Taipei, Taiwan.

[26] Shoop C, Binnie C (1979) The effects of age on the visual perception of speech. Scandinavian Audiology 8: 3–8. 515681 10.3109/01050397909076295

[27] Cienkowski KM, Carney AE (2002) Auditory-visual speech perception and aging. Ear Hear 23: 439–449. 12411777 10.1097/00003446-200210000-00006

[28] Sommers MS, Tye-Murray N, Spehar B (2005) Auditory-visual speech perception and auditory-visual enhancement in normal-hearing younger and older adults. Ear Hear 26: 263–275. 15937408 10.1097/00003446-200506000-00003

